# Peripheral CD4^+^CD25^hi^CD127^low^ regulatory T cells are increased in patients with gastrointestinal cancer

**DOI:** 10.1186/s12876-023-02798-0

**Published:** 2023-05-20

**Authors:** Junlan Qiu, Weiqiang Shi, Jin Zhang, Qinqin Gao, Lin Feng, Zhixiang Zhuang

**Affiliations:** 1 Department of Oncology, Suzhou Science and Technology Town Hospital, Suzhou, Jiangsu, 215153 China; 2 Department of Pathology, The First Affiliated Hospital of Soochow University, Suzhou, Jiangsu, 215006 China; 3 Department of Pathology, Suzhou Science and Technology Town Hospital, Suzhou, Jiangsu, 215153 China; 4 Institute for Fetology, The First Affiliated Hospital of Soochow University, Suzhou, Jiangsu, 215006 China; 5 Department of Oncology, The Second Affiliated Hospital of Soochow University, Suzhou, Jiangsu, 215004 China

**Keywords:** Gastrointestinal cancer, CD4^+^CD25^hi^CD127^low^ Tregs, IL-10, TGF-β1

## Abstract

**Background:**

Regulatory T cells (Tregs) play an important role in regulation of immune response and immunologic tolerance in cancer. Gastrointestinal cancer is still a leading cause of cancer-related death in the world. This study aimed to detect Tregs in patients with gastrointestinal cancer.

**Methods:**

In this study, 45 gastric cancer patients, 50 colorectal cancer patients and 50 healthy controls were enrolled. Flow cytometry was used to detect CD4^+^CD25^hi^CD127^low^ Tregs, CD4^+^CD25^hi^, and CD4^+^ cells in peripheral blood. Cytokine interleukin-10 (IL-10) and transforming growth factor-β1 (TGF-β1) in peripheral blood and in the supernatant of Tregs cultures were measured by enzyme linked immunosorbent assay.

**Results:**

Compared with healthy controls, the levels of CD4^+^CD25^hi^CD127^low^ Tregs and CD4^+^CD25^hi^ cells increased significantly in patients with gastrointestinal cancer. Patients with gastrointestinal cancer also showed a significantly increased levels of IL-10 and TGF-β1 in both peripheral blood and CD4^+^CD25^hi^CD127^low^ Tregs culture medium.

**Conclusion:**

The present study firstly demonstrated that gastrointestinal patients have a compromised immune status where the CD4^+^CD25^hi^CD127^low^ Tregs, as well as levels of IL-10 and TGF-β1 are elevated. The data offered new information for understanding the immunological features of gastrointestinal patients, as well as provided new insights into approaches to develop new immunotherapies for patients with gastrointestinal cancer.

## Background

The gastrointestinal (GI) carcinoid tumors are used collectively to refer to cancers of the digestive tract and includes gastric, pancreas, colorectal and anal cancers, of which gastric cancer (GC) and colorectal cancer (CRC) accounts for the vast majority. GI cancers are common malignancies and one of the leading causes of cancer deaths worldwide. For instance, GC is the third most common cancer in men and fifth in women, and is still a leading cause of cancer-related death [1, 2] and CRC is the second most common cancer diagnosed in women and third most in men [3], which imposes a considerable health burden globally. In recent years, with the development of tumor immunology research, the inability of the immune system to eradicate tumor is one of the fundamental hallmarks of cancer [4–6]. Tumors exploit multiple mechanisms to suppress host immunity and promote immune evasion. Immune escape as well as immune checkpoints play a key role in the occurrence, development, invasion, and metastasis of numerous cancers [4–6].

Many studies have reported the roles of regulatory T cells (Tregs) in helping tumors evade immune surveillance [7, 8]. Tregs, a T lymphocyte subset, is of great importance in the regulation of tumor immunity and autoimmunity promoting tumor progression by suppressing effective antitumor immunity [7, 8]. Naturally occurring Tregs represent about 5% of the CD4^+^ cell subset in peripheral blood and constitutively express high levels of the high-affinity CD25. Recently, Tregs have been identified as CD4^+^CD25^hi^ T cells with low levels of CD127 expressing a CD4^+^CD25^hi^ CD127^low^ cell surface phenotype [9, 10]. Tregs can also induce an immunosuppressive microenvironment that blocks successful tumor immunotherapy [11, 12]. Tregs can exert their immunosuppressive effects by stimulating the secretion of transforming growth factor-β1 (TGF-β1) and interleukin-10 (IL-10) [13–15]. TGF-β1 is a potent suppressor of the immune system, which is also central to immune suppression within the tumor microenvironment [13]. Recent studies revealed that TGF-β1 promoted the progression, invasion, and metastasis in GI cancer, and may be also associated with the prognosis [16, 17]. IL-10 is recognized for inhibiting the activation and effector function of T cells, monocytes, and macrophages, and thus, it is a multifunctional cytokine with diverse effects in immunity and cancer [18].

Although accumulating studies had reported that a significant increase in CD4^+^CD25^hi^CD127^low^ sub-population in some tumors [9, 19], the information of Tregs in GI cancer is very limited. Detecting peripheral CD4^+^CD25^hi^CD127^low^ Tregs and related cytokines would help to comprehensively evaluate and understand the function of patient immunity from multiple perspectives, so as to guide clinical practice to conduct a more global pre-evaluation. Therefore, in this study, we aimed to detect CD4^+^CD25^hi^CD127^low^ Tregs, TGF-β1 and IL-10 levels to evaluate the immunosuppression status in patients with GI cancer, would provide useful information for the development of effective immunotherapies against GI cancer.

## Methods

### Sample collection

Gastric cancer (GC, *N* = 45) and colorectal cancer (CRC, *N* = 50) were recruited from at the Department of Oncology, Affiliated Suzhou Science and Technology Town Hospital of Nanjing Medical University from July 2016 to July 2020. The following criteria had to be met for inclusion: 1) Diagnosis with fiber gastroscopy or colonoscopy; 2) No radiochemotherapy, immune modulator therapy, glucocorticoids, or nonsteroidal drug therapy within two months; 3) Complete clinical information. Patients who met one of the following criteria would be excluded: 1) Received radiochemotherapy, immune modulator therapy, glucocorticoids, or nonsteroidal drug therapy within two months; 2) Patients with acute infection, long-term chronic inflammation, or other immune system diseases; 3) No pathological diagnosis; 4) Incomplete clinical information. Simultaneously, 50 healthy people as determined by physical examination were selected for the health control group (HC, *N* = 50). The pathological stage of GC and CRC was classified into I, II, III and IV stages according to the 8th edition of the UICC/AJCC TNM staging system. TMN stage was composed of depth of tumor invasion (T), regional lymph node metastasis (N) and distant metastasis (M). In this study, the patients were divided into an early stage (I + II) group and a late‐stage (III + IV) group. In addition, according to the World Health Organization (WHO) histopathological classification of gastrointestinal cancer, tumor differentiation was also categorized into three grades: poor, moderate, and high. The clinical characteristics of all participants were detailed in Table 1. Eight to ten milliliters of early morning peripheral venous blood from fasted participants were collected with heparin anticoagulation vacuum blood collection tubes, and then was fully mixed with EDTA for anticoagulation. The samples were then stored at room temperature and stained within 6 h. All the research subjects voluntarily participated in this project and signed an informed consent form. Additionally, the study was approved by the ethics committee of Suzhou Hospital Affiliated with Nanjing Medical University.

**Table 1:** The characteristics of patients with gastric cancer or colorectal cancer. N, number of participants

Characteristics	Healthy control		Gastric cancer (GC)		Colorectal cancer (CRC)	
	**N**	**%**	**N**	**%**	**N**	**%**
**Sex**						
Male	26	52.00	23	51.11	28	56.00
Female	24	48.00	22	48.89	22	44.00
**Age**						
< 60	32	64.00	28	62.22	37	74.00
≥ 60	18	36.00	17	37.78	13	26.00
**Differentiation**						
Poor	**/**	**/**	24	53.33	11	22.00
Moderate	**/**	**/**	18	40.00	34	68.00
High	**/**	**/**	3	6.67	5	10.00
**Recurrence and Metastasis**						
yes	**/**	**/**	19	42.22	12	24.00
no	**/**	**/**	26	57.78	38	76.00
**Stage**						
Early (Stage I & II)	**/**	**/**	18	40.00	33	66.00
Late (Stage III & IV)	**/**	**/**	27	60.00	17	34.00
**Habits**						
Ever smoker	4	8.00	8	17.78	6	12.00
Ever Drinker	5	10.00	7	15.56	10	20.00
No habit	41	82.00	30	66.67	34	16.00

### Flow cytometric analysis

To prepare the samples for flow cytometry, 100 μl EDTA-anticoagulated whole blood was collected in a dedicated flow test tube. The cells were stained in vitro using the trypan blue exclusion method, and the percentage of living cells had to be greater than 99%. Appropriate amounts of fluorescently labeled monoclonal antibodies (anti-CD4-FITC, anti-CD25-APC, and anti-CD127-PE) were then added, and the samples were mixed. Cells were incubated at room temperature for 15 min in the dark. Then, red blood cells were lysed, incubated in the dark for 12 min, and then centrifuged at 1500 rpm for 5 min. The supernatants were discarded, and the resulting cell pellet was subsequently resuspended in 1 ml phosphate-buffered saline (PBS). The cells were centrifuged again for 5 min at 1500 rpm, after which the supernatant was discarded. Finally, 400 μl PBS was added to the final cell pellet, and the cells were examined on a flow cytometer (FACSCalibur, BD Biosciences, California, USA). The control tubes were stained using the same procedure as that used for the experimental group. The presence of CD4^+^, CD4^+^CD25^hi^ and CD4^+^CD25^hi^CD127^low^ cells was then analyzed using FACSDiva analysis software (BD Biosciences, California, USA). The anti-CD4-FITC, anti-CD25-APC, and anti-CD127-PE monoclonal antibodies and appropriate negative controls (mouse IgG1-FITC, IgG1-APC, and IgG1-PE) were all obtained from BD Biosciences-Pharmingen.

### Cytokine detection

Enzyme linked immunosorbent assay (ELISA) was used to determine the levels of IL-10 and TGF-β1 in serum from peripheral blood. Briefly, 96-well plates were washed three times with an automatic plate washer. After drying, analytical buffer and standard series solutions were added to the wells, and 60 μl of analytical buffer was sequentially dripped into the remaining wells. The enzyme-conjugated antibodies were dripped into each well, and then the plates were incubated for 1.5 h at 22 °C. Then, the wells were washed 3 times (each time lasts 5 min) with PBS. Each well was covered with 80 μl of horseradish peroxidase (HRP)-streptavidin solution. The plate was incubated at room temperature for 1.5 h and washed 3 times before drying. Then, 100 μl chromogenic agent was dripped into the wells, and the plates were incubated at 20 °C for 15 min. The optical density was measured at 450 nm with a microplate reader, and the IL-10 and TGF-β1 concentrations were calculated.

For IL-10 and TGF-β1 in Tregs culture medium, Ficoll density gradient centrifugation was used to obtain peripheral blood mononuclear cells (PBMCs) from 5 ml of peripheral blood. Anti-CD4-FITC, anti-CD25-APC, and anti-CD127-PE fluorescent antibodies were used to label the cells. High purity CD4^+^CD25^hi^CD127^low^ Tregs were separated by flow cytometry. Tregs were cultured in RPMI-1640 medium containing 10% fetal bovine serum, and 1 × 10 [6] were added to each well of 96-well plate coated with CD3 antibody. Anti-CD28 monoclonal antibody (1 µg/ml) and IL-2 (500 pg/ml) were added to each well. After being cultured for 3 and 5 days, the supernatants were collected for measuring IL-10 and TGF-β1 levels via ELISA.

### Statistical analysis

Data were expressed as mean ± standard error of the mean (SEM). Data were analyzed by Student’s t test (GraphPad Software, San Diego, CA, USA). Results with a *P*-value < 0.05 were considered statistically significant.

## Results

### Demographic and clinical characteristics of gastrointestinal patients

In this study, there were 23 males and 22 females between 32–72 years old (57.8 ± 6.9) with gastric cancer. 50 patients with colorectal cancer we selected included 28 males and 22 females, who were between 47 and 73 (57.6 ± 3.2) years old (Table 1). Persons of healthy control group were selected from the physical examination center.

### CD4^+^CD25^hi^CD127^low^ Tregs were up-regulated in patients with gastrointestinal cancer

**Fig. 1:**
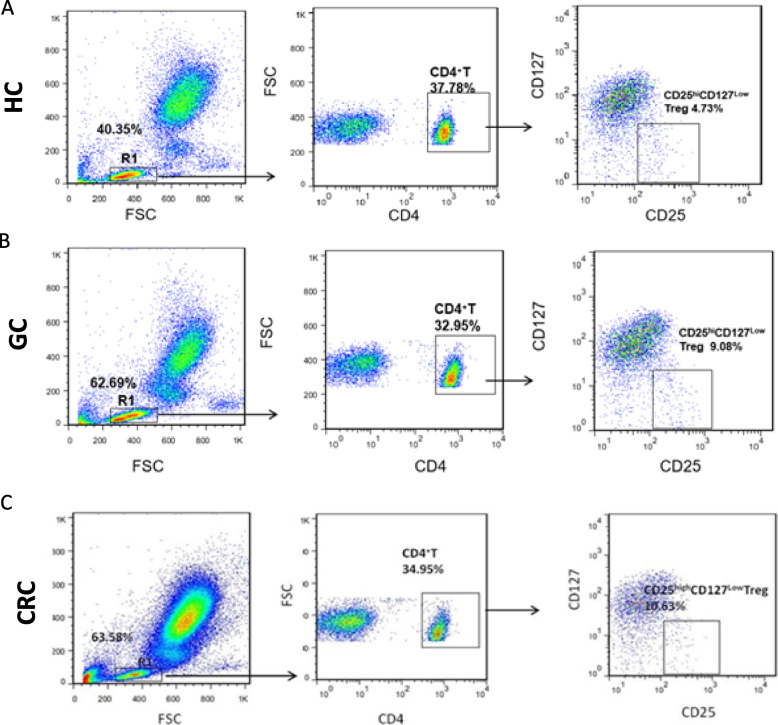
Flow cytometry scatter plot of patients with gastrointestinal cancer, and healthy controls. Left: R1, a scatter plot of lymphocytes; middle: CD4^+^ T cells were gated by R1; right: CD4^+^CD25^hi^CD127^low^ Tregs were set up with CD4^+^. HC, healthy control; GC, gastric cancer; CRC, colorectal cancer

**Fig. 2:**
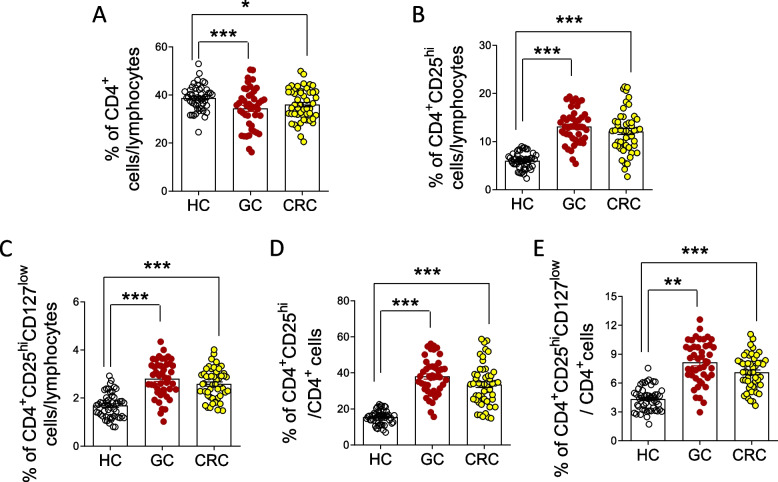
CD4^+^CD25^hi^CD127^low^ Tregs were up-regulated in patients with gastrointestinal cancer. **A** Percentage of CD4^+^ cells amongst patients and healthy controls. **B** Percentage of CD4^+^CD25hi cells amongst patients and healthy controls. **C** Percentage of CD4^+^CD25^hi^CD127^low^ cells amongst patients and healthy controls. **D** Percentage of CD4^+^CD25^hi^ cells amongst CD4^+^ cells. **E** Percentage of CD4^+^CD25^hi^CD127^low^ cells amongst CD4^+^ cells. HC, healthy control (*N* = 50); GC, gastric cancer (*N* = 45); CRC, colorectal cancer (*N* = 50). N, number of patients or healthy controls. Data were presented as means ± SEM and analyzed by Student’s t test. *, *P* < 0.05; **, *P* < 0.01; ***, *P* < 0.001

To evaluate the immunosuppression of GI patients, CD4^+^CD25^hi^CD127^low^ Tregs in the peripheral blood was detected by flow cytometry. A flow cytometry scatter plot shows the percentages of peripheral CD4^+^, CD4^+^CD25^hi^, and CD4^+^CD25^hi^CD127^low^ Tregs in patients with GI or healthy controls (Fig. 1A-C). As shown in Fig. 2A-C and Table 2, compared with the healthy controls (HC), the proportion of peripheral CD4^+^CD25^hi^ and CD4^+^CD25^hi^CD127^low^Tregs increased remarkably in the GC group (CD4^+^CD25^hi^: GC *vs.* HC; 13.11 ± 0.58 *vs.* 6.01 ± 0.33, *P* = 0.0008; CD4^+^CD25^hi^CD127^low^: GC *vs.* HC; 2.81 ± 0.36 *vs.*1.68 ± 0.14, *P* = 0.0007) and CRC patients group (CD4^+^CD25^hi^: CRC *vs.* HC; 12.09 ± 0.69 *vs.* 6.01 ± 0.33, *P* = 0.0009; CD4^+^CD25^hi^CD127^low^: CRC vs HC; 2.56 ± 0.18 *vs.* 1.68 ± 0.14, *P* = 0.0007), while the percentages of peripheral CD4^+^ cells decreased in the GC (GC *vs.* HC; 33.95 ± 1.27 *vs.* 38.72 ± 0.73, *P* = 0.0009) or CRC patients (CRC *vs.* HC; 35.94 ± 1.01 *vs.* 38.72 ± 0.73, *P* = 0.0233) (Fig. 2A-C). Notably, the ratio of the expanded subset of the CD4^+^CD25^hi^ cells, or CD4^+^CD25^hi^ CD127^low^ Tregs to CD4^+^ cells was further determined; GC and CRC patients demonstrated an increase in the ratio as compared to healthy donors (Fig. 2D-E).

**Table 2:** Percentages of peripheral T lymphocyte subsets in the three groups. HC, healthy control; GC, gastric cancer; CRC, colorectal cancer. Data were presented as means ± SEM and analyzed by Student’s t test. N, number of participants; *, *P* < 0.05; ***, *P* < 0.001

Group	N	CD4^+^	CD25^hi^	CD4^+^CD25^hi^ CD127^low^	
**HC**	50	38.72 ± 0.73	6.01 ± 0.33	1.68 ± 0.14	
**GC**	45	33.95 ± 1.27***	13.11 ± 0.58***	2.81 ± 0.36***	
**CRC**	50	35.94 ± 1.01*	12.09 ± 0.69***	2.56 ± 0.18***	

### Increases of IL-10 and TGF-β1 in patients with gastrointestinal cancer

**Fig. 3:**
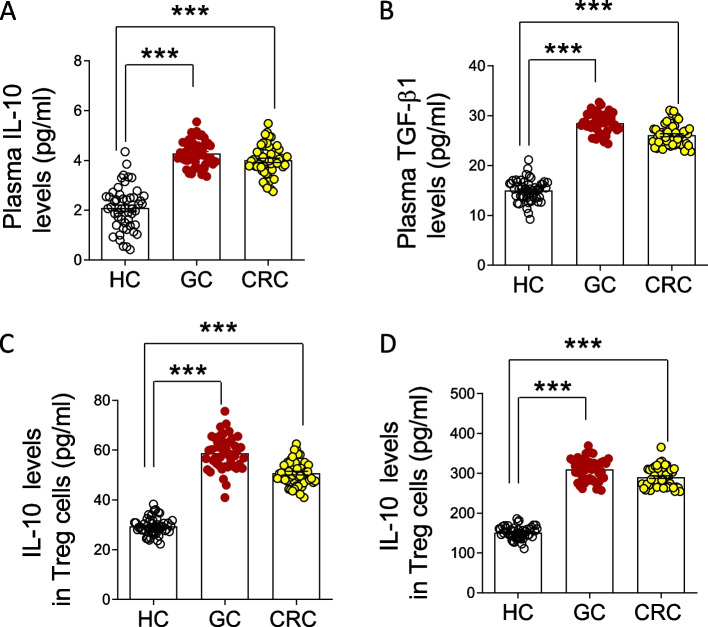
Increases of IL-10 and TGF-β1 in patients with gastrointestinal cancer. **A** and **B** IL-10 and TGF-β1 concentration in the peripheral blood of patients and healthy controls. **C** and **D** IL-10 and TGF-β1 concentrations in the Tregs supernatant. HC, healthy control (*N* = 50); GC, gastric cancer (*N* = 45); CRC, colorectal cancer (*N* = 50). N, number of patients or healthy controls. Data were presented as means ± SEM and analyzed by Student’s t test. ***, *P* < 0.001

Because Tregs can exert their immunosuppressive effects through stimulating the production of TGF-β1 and IL-10, the concentrations of the two cytokines in peripheral blood were determined to further assess the immunosuppressive condition of patients. Compared with those of the healthy controls, IL-10 and TGF-β1 concentrations increased significantly in the peripheral blood of the GC (IL-10: GC *vs.* HC; 4.28 ± 0.09 *vs.* 2.09 ± 0.15, *P* = 0.0006; TGF-β1: GC *vs.* HC; 28.51 ± 0.34 *vs.* 15.02 ± 0.31, *P* = 0.0008) and CRC patients (IL-10: CRC vs. HC; 4.01 ± 0.12 *vs.* 2.09 ± 0.15, *P* = 0.0008; TGF-β1: CRC *vs.* HC; 26.07 ± 0.29 *vs.* 15.02 ± 0.31, *P* = 0.0005) (Fig. 3A-B and Table 3).

**Table 3:** Comparison of IL-10 and TGF-β1 in the peripheral blood of the three groups. HC, healthy control; GC, gastric cancer; CRC, colorectal cancer. Data were presented as means ± SEM and analyzed by Student’s t test. N, number of participants; ***, *P* < 0.001

**Group**	**N**	**IL-10** (pg/ml)	**TGF-β1 (**pg/ml**)**
**HC**	50	2.09 ± 0.15	15.02 ± 0.31
**GC**	45	4.28 ± 0.09***	28.51 ± 0.34***
**CRC**	50	4.01 ± 0.12***	26.07 ± 0.29***

To further determined the relation of the two cytokines and CD4^+^CD25^hi^CD127^low^ Tregs, the concentrations of IL-10 and TGF-β1 in cultured CD4^+^CD25^hi^CD127^low^ Tregs were also detected by ELISA. Compared with the healthy group, the IL-10 and TGF-β1 concentrations in CD4^+^CD25^hi^CD127^low^ Tregs increased significantly in the peripheral blood of the GC and CRC patients (Fig. 3C-D and Table 4).

**Table 4:** Comparison of IL-10 and TGF-β1 in Treg cells supernatant of the three groups. HC, healthy control; GC, gastric cancer; CRC, colorectal cancer. Data were presented as means ± SEM and analyzed by Student’s t test. N, number of participants; ***, *P* < 0.001

**Group**	**N**	**IL-10 (**pg/ml**)**	**TGF-β1 (**pg/ml**)**
**HC**	50	29.35 ± 0.48	151.21 ± 2.18
**GC**	45	58.75 ± 1.02***	309.61 ± 4.06***
**CRC**	50	58.78 ± 0.69***	290.39 ± 3.57***

## Discussion

Although concentrated research efforts focusing on GI cancer have been emerging, the effects of traditional surgery, chemotherapy, and radiotherapy are limited, and a great number of patients with GI cancer still develop local recurrence and distant metastasis after receiving these treatments. Because the current immunotherapy for GI cancer remains inadequate, there is a great need to obtain useful information for developing novel therapeutic targets in GI cancer. In the current study, we found that, compared with healthy controls, CD4^+^CD25^hi^CD127^low^ Tregs, CD4^+^CD25^hi^ cells, and the related cytokines, IL-10 and TGF-β1, were significantly increased in patients with GI cancer.

Immune response in cancer development, recurrence, and especially treatment is of great importance. CD4^+^CD25^hi^CD127^low^ Tregs participate in immune self-tolerance and immune homeostasis regulation during the pathophysiological immune response [20]. Accumulating studies had demonstrated that a large number of CD4^+^CD25^hi^CD127^low^ Tregs infiltrate into various types of tumors in humans, including gastrointestinal tract cancer [21–24]. Furthermore, Tregs are generally regarded as key immunomodulators that maintain immune tolerance and induce an immunosuppressive microenvironment [20, 23]. To evaluate the immunosuppressive microenvironment of GI cancer patients, we used flow cytometry to detect peripheral CD4^+^ cells and CD4^+^CD25^hi^CD127^low^ Tregs, and the results demonstrated that CD4^+^CD25^hi^CD127^low^ Tregs and CD4^+^CD25^hi^ cells significantly increased in patients with GI cancer compared with the healthy group.

CD4^+^CD25^hi^CD127^low^ Tregs can regulate their suppressive effects through stimulating the production of IL-10 and TGF-β1 [13–15]. IL-10 is closely related to the development and prognosis of GI cancer [25–27]. IL-10 distinctly modulates GI cancer cell behavior, as a result of distinct proteolytic profiles that impacted cell invasion and angiogenesis, and high IL-10 expression has been shown to be significantly associated with poorer prognosis in GI patients [26, 27]. TGF-β1 secretion by tumor-associated macrophages promotes proliferation, invasion, and metastasis of GI cancer [28, 29]. In combination, high expressions of IL-10 and TGF-β1 promotes the development of GI cancer, tumor metastasis, and leaded to worse prognosis. This study also measured the concentrations of IL-10 and TGF-β1 in peripheral blood and in cultured CD4^+^CD25^hi^CD127^low^ Tregs in patients with GI cancer, and found the two cytokines were significantly higher in patients with GI cancer than that in healthy controls. These data together suggested that the increased CD4^+^CD25^hi^CD127^low^ Tregs, accompanied with higher IL-10 and TGF-β1 in patients with GI cancer could may play a role in the development and prognosis of GI cancer.

Additionally, our previous research proved that CD4^+^CD25^hi^CD127^low^ Tregs in peripheral blood had great value for the selection of appropriate individualized treatment options for patients with non-small cell lung cancer [19]. What’s more, the expression of PD-L1 on the surface of peripheral CD4^+^CD25^hi^CD127^low^ Tregs was significantly increased in patient with primary hepatocellular carcinoma, especially in patients with advanced disease, suggesting that the high PD-L1 expression on the surface of CD4^+^CD25^hi^CD127^low^ Tregs may be related to tumor progression and patient prognosis [24]. Therefore, determining the PD-L1 expression on the surface of CD4^+^CD25^hi^CD127^low^ Tregs in GI cancer would improve our understanding of the relationship between CD4^+^CD25^hi^CD127^low^ Tregs and the treatment or prognosis of GI patients. Such studies are currently the focus of intensive ongoing investigation in our laboratory.

## Conclusions

In conclusion, the current findings firstly indicated that GI patients have a compromised immune status where the CD4^+^ cell are reduced and the CD4^+^CD25^hi^CD127^low^ Tregs are elevated. Peripheral CD4^+^CD25^hi^CD127^low^ Tregs could be used for the evaluation of immunosuppressive status of patients with GI cancer and might assist in appraising the potential efficacy of tumor immunotherapies in GI cancer. Therapies targeted to CD4^+^CD25^hi^CD127^low^ Tregs, IL-10 and TGF-β1 in patients with GI cancer would be of great clinical significance in the individualization and optimization of treatment in the future.

## Data Availability

The datasets used and/or analyzed during the current study are available from the corresponding author upon reasonable request.

## Abbreviations

Tregs: Regulatory T cells
IL-10: Cytokine interleukin-10
TGF-β1: Transforming growth factor-β1
GI: The gastrointestinal carcinoid tumors
GC: Gastric cancer
CRC: Colorectal cancer
